# Efficacy prediction of acupuncture treatment for migraine without aura based on multimodal MRI: A study protocol

**DOI:** 10.3389/fneur.2022.953921

**Published:** 2022-10-10

**Authors:** Shirui Cheng, Xinyue Zhang, Huabin Zheng, Nannan Jiang, Jun Zhou, Xinling Li, Yu Fang, Xiaopeng Huang, Jingtao Liang, Tao Yin, Fanrong Liang, Fang Zeng, Zhengjie Li

**Affiliations:** ^1^Acupuncture and Tuina School, Chengdu University of Traditional Chinese Medicine, Chengdu, China; ^2^Acupuncture and Brain Research Center, Chengdu University of Traditional Chinese Medicine, Chengdu, China; ^3^Hospital of Chengdu University of Traditional Chinese Medicine, Chengdu, China

**Keywords:** acupuncture, migraine without aura, efficacy prediction, magnetic resonance imaging, resting state

## Abstract

**Introduction:**

Acupuncture is an effective and safe therapy for patients with migraine without aura (MwoA), but only 41–59% of patients show improvement with this treatment. Screening positive responders to acupuncture treatment for MwoA can ensure that healthcare resources can be appropriately targeted to specific patients who would most benefit. The objective of this study is to determine whether the structure and functional activity in certain brain regions can predict analgesia response in patients with MwoA who receive acupuncture treatment.

**Methods and analysis:**

A total of 72 patients with MwoA and 72 healthy controls (HCs) will be enrolled in this study. Resting-state structural and functional magnetic resonance imaging (MRI) data will be collected from each participant at baseline. Patients with MwoA will undergo 12 sessions of acupuncture treatment for 8 weeks, twice per week in the first 4 weeks and once per week for the last 4 weeks. The follow-up will be 12 weeks. The number of days with migraine, frequency of migraine attacks, and average visual analog scale scores will be recorded in detail at weeks 0, 4, 8, 12, and 16 and at the end of follow-up (week 20). The positive response rate will be calculated as the proportion of patients with ≥50% reduction in the number of migraine days during follow-up compared with baseline. Machine learning methods will be applied to classify patients with MwoA and HCs and predict patients with response or non-response to acupuncture treatment based on multimodal MRI parameters, such as gray matter volume, regional homogeneity, amplitude of low-frequency fluctuation, fractional anisotropy, and mean diffusivity.

**Discussion:**

This study aims to establish brain structural and functional characteristics that can identify patients with MwoA who will derive analgesia benefits from acupuncture treatment.

**Trial registration number:**

http://www.chictr.org.cn/showproj.aspx?proj=65443, identifier: ChiCTR2100042915.

## Introduction

Migraine is a common neurologic disorder characterized by recurrent attacks of disabling headaches that are often accompanied by nausea, vomiting, photophobia, and phonophobia (1). Migraine is the 7th leading cause of time spent disabled worldwide (2), with an estimated 1-year prevalence of 15% in the general population (3). At present, there are no satisfactory treatments for migraine (4–6). Acupuncture, an important component of traditional Chinese medicine, is recommended by the World Health Organization as an alternative and complementary therapy for migraine. Many randomized controlled trials and systematic reviews have demonstrated that acupuncture is safe and cost-effective with comparable efficacy to prophylactic drugs for migraine and can reduce headache intensity, frequency of migraine attacks, and number of migraine days (7–10).

However, only 41–59% of patients with migraine without aura (MwoA) experience symptom improvement with acupuncture treatment (9). This is associated not only with clinical but also with socioeconomic challenges. Therefore, identifying characteristics that can be used to predict migraineurs' response to acupuncture is important to improve the efficacy of the treatment and provide individualized therapy to patients who would most benefit.

Personalized medicine can be achieved by conducting clinical trials, assessing clinical outcomes, analyzing numerous characteristics, and identifying common and unique predictors of treatment outcomes. Previous studies have reported that patients' expectations, educational background, and treatment preferences influence the clinical efficacy of acupuncture for diseases characterized by pain (11–15). However, the questionnaires used in these studies were subjective and qualitative. Recently, with the development of neuroimaging techniques and artificial intelligence, magnetic resonance imaging (MRI) data have been used to predict the clinical efficacy of various interventions, including pharmacologic treatments (16), cognitive behavioral therapy (17), and placebo treatments (18), among others. These studies often provide more objective and reliable predictions than questionnaires.

Patients with MwoA show widespread structural and functional changes in cortical and subcortical brain regions (19, 20). Acupuncture can potentially alleviate brain dysfunction in migraineurs in a specific way (21–24). Some studies have used either structural or functional parameters of different brain regions to predict the efficacy of verum or sham acupuncture treatment for chronic pain diseases (18, 25–27), demonstrating that objective neuroimaging parameters can be used to predict the analgesic efficacy of acupuncture treatment in patients with migraine.

The aim of this study is to identify brain features detected by multimodal MRI that can be used to predict the response to acupuncture treatment. To this end, multimodal MRI indicators from patients with MwoA and healthy controls (HCs) will be analyzed, then an advanced multivariate pattern analysis (MVPA) based on support vector classification (SVC) will be conducted through training, validation, and testing approach to classify migraineurs and HCs. Responders and non-responders of acupuncture treatment for patients with MwoA will be identified by the clinical outcomes obtained before vs. after treatment. The efficacy of acupuncture will be predicted by support vector regression (SVR) based on multimodal MRI data detected at baseline.

## Methods and analysis

### Study design

**Figure 1:**
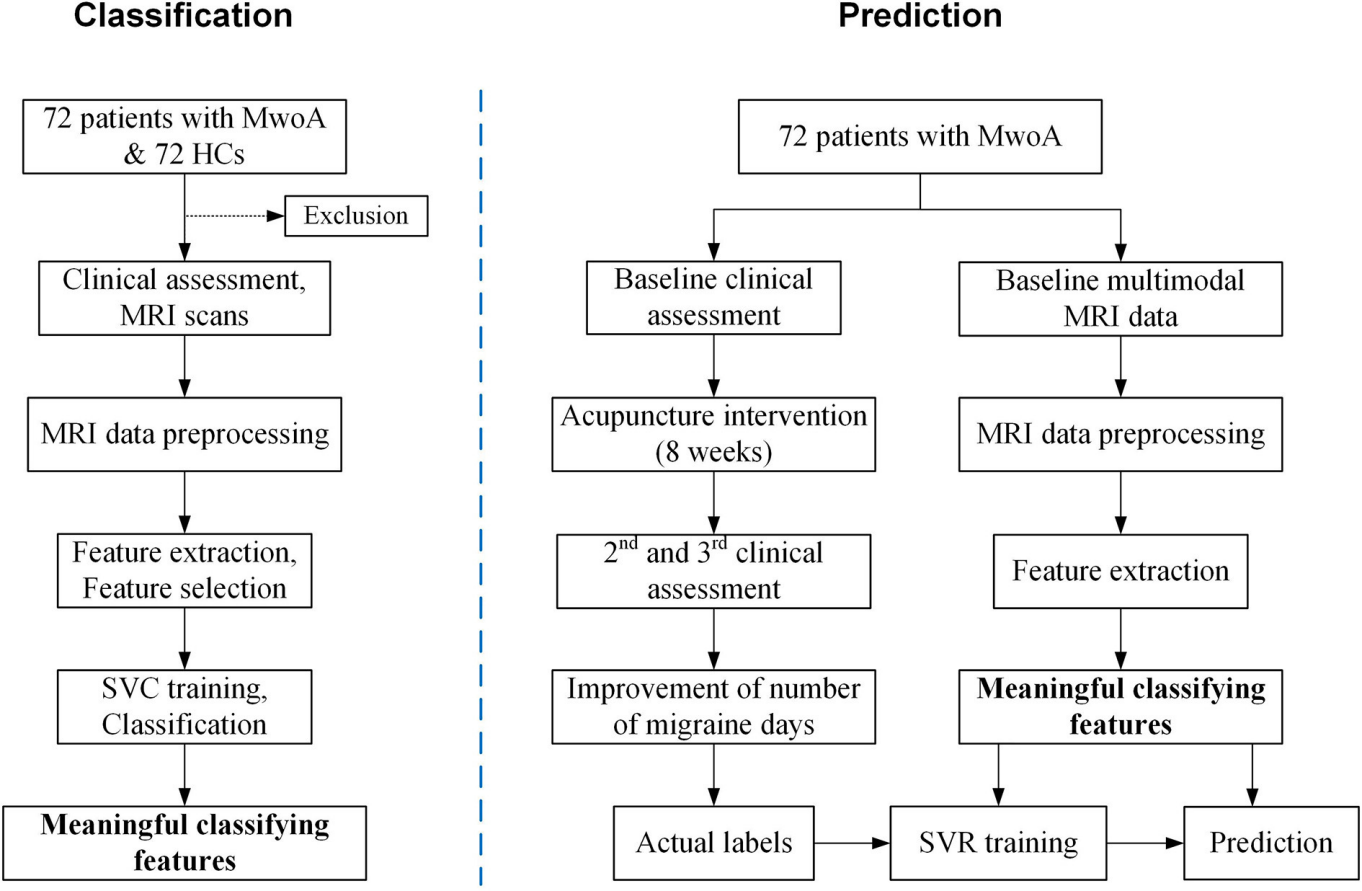
Study flowchart. The flowchart of this study.

This non-randomized clinical trial will enroll 72 patients with MwoA and 72 with HCs. The study will last 24 weeks including a 4-week baseline period; for migraineurs, this will be followed by an 8-week treatment period and a 12-week follow-up period (Table 1). Structural and functional MRI scans will be performed at baseline. Clinical outcomes will be evaluated after treatment and during the follow-up. Figure 1 shows a flow diagram of the study. This protocol is reported in accordance with Consolidated Standards of Reporting Trials and Standards for Reporting Interventions in Clinical Trials of Acupuncture. The protocol was approved by the Institutional Review Board and Ethics Committee of the Hospital of Chengdu University of Traditional Chinese Medicine (approval no. 2020KL-058) and is registered with the Chinese Clinical Trial Registry (registration no. ChiCTR2100042915).

**Table 1:** Standard protocol items: recommendations for interventional trials (SPIRIT) schedule of the trial.

**Study period**							
**Items**	**Screening**	**Baseline**	**Treatment**		**Follow-up**		
**Timepoint**	**week 4**	**week 0**	**week 4**	**week 8**	**week 12**	**week 16**	**week 20**
**Enrolment**	
Eligibility screen	**×**						
Informed consent	**×**						
**Intervention**	
Acupuncture			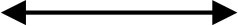		
**Assessments**	
Headache diary	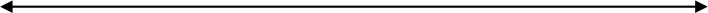						
Number of days with headache	**×**	**×**	**×**	**×**	**×**	**×**	**×**
Frequency of headache attacks	**×**	**×**		**×**			**×**
Average VAS score	**×**	**×**	**×**	**×**	**×**	**×**	**×**
SAS score		**×**	**×**	**×**	**×**	**×**	**×**
SDS score		**×**	**×**	**×**	**×**	**×**	**×**
Use of NSAIDs	**×**	**×**		**×**			**×**
**Participants safety**	
Laboratory test		**×**		**×**			
MRI scan		**×**					
Adverse events	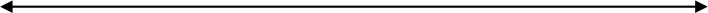						

This is a clinical trial that includes a 4-week baseline period, 8-week treatment period, and 12-week follow-up period. In the baseline period, eligible patients with MwoA will be screened and recruited. Before the treatment, patients will undergo a physical examination and structural and functional MRI scans. During the 8-week treatment period, patients will undergo 12 acupuncture sessions. The clinical outcome assessment will include the number of days with headache, frequency of migraine attacks, average VAS score, SAS score, SDS score, and use of non-steroidal anti-inflammatory drugs. In addition, patients will keep a headache diary in which they will record each migraine attack experienced during the trial. The physical examination, which will include routine blood biochemistry, will also be performed at the end of the treatment period to evaluate the potential risks associated with acupuncture. Adverse events occurring at any time during the trial will be recorded.

a. VAS, Visual Analog Scale; SAS, Self-rating Anxiety Scale; SDS, Self-rating Depression Scale; NSAIDs, Non-steroidal Anti-inflammatory Drugs. Laboratory test: including routine examination of blood, urine and stool, blood biochemical test (including liver and kidney function), and electrocardiogram examination.

### Participants

Seventy-two patients with MwoA and 72 with HCs will be recruited for this study. Inclusion criteria for patients with MwoA are as follows: (1) aged 18–60 years; (2) right handed; (3) diagnosed with MwoA according to the 2018 International Classification of Headache Disorders (ICHD-3) (1); (4) have a history of migraine for over 6 months; (5) have had migraine attacks 3–15 times per month in the past 3 months; (6) did not take prophylactic headache medicine or receiving acupuncture treatment in the previous 3 months; and (7) signed the informed consent form.

Exclusion criteria were as follows: (1) suffering from psychiatric, neurologic, cardiovascular, respiratory, or renal illness; (2) suffering from any other chronic pain conditions or have a history of head trauma with loss of consciousness; (3) alcohol or drug abusers; (4) are pregnant or lactating; (5) have acupuncture contraindications such as bleeding tendency; and (6) have MRI contraindications such as claustrophobia.

Inclusion criteria for HCs are as follows: (1) aged 18–60 years; (2) right handed; and (3) free of any diseases. HCs will be excluded if they meet the following criteria: (1) have drug or alcohol addiction; (2) have contraindications for MRI; and (3) pregnant or lactating.

### Participant recruitment

All MwoA patients and HCs will be recruited as outpatients and inpatients of the Neurology Department of the Hospital of Chengdu University of Traditional Chinese Medicine or from the community through advertising from March 2021 to December 2023.

### Blinding

This trial follows the principle of separation of researchers, evaluators, and statistical analysts.

### Interventions

**Figure 2:**
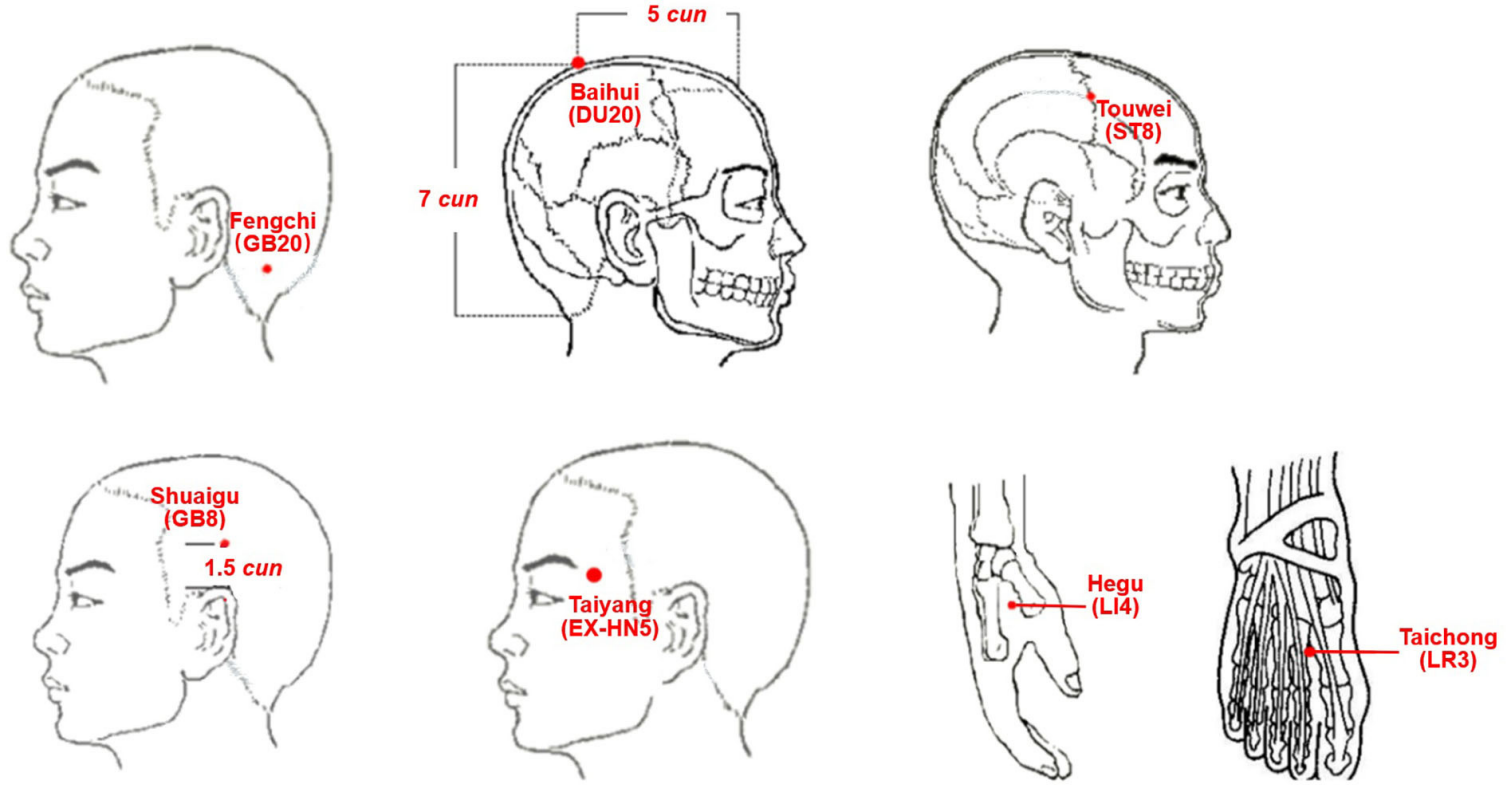
Location of Acupoints. DU20 (*Baihui*): on the head, five *cun* directly above the midpoint of the anterior hairline or at the midpoint of the line connecting the apexes of the two auricles. GB20 (*Fengchi*): on the nape, below the occiput in the depression between the upper portion of the trapezius and sternocleidomastoid. ST8 (*Touwei*): at the lateral side of the head, within 0.5 *cun* of the hairline at the corner of the forehead, and 4.5 *cun* lateral to the median line of the head. GB8 (*Shuaigu*): on the head above the apex of the auricle and SJ-20 (*Jiaosun*), within 1.5 *cun* of the hairline. EX-HN5 (*Taiyang*): in the region of the temples, in the depression about one finger-width posterior to the midpoint between the lateral end of the eyebrow and outer canthus. LI4 (*Hegu*): on the dorsum of the hand, between the 1st and 2nd metacarpal bones, at the midpoint of the 2nd metacarpal bone on the radial side. LR3 (*Taichong*): on the dorsum of the foot, in the depression proximal to the first metatarsal space.

HCs will not receive any treatment during the trial. All patients with MwoA will receive the same acupuncture treatment procedure. *Baihui* (GV20), bilateral *Fengchi* (GB20), bilateral *Touwei* (ST8), bilateral *Shuaigu* (GB8), bilateral *Taiyang* (EX-HN5), bilateral *Hegu* (LI4), and bilateral *Taichong* (LR3) will be targeted by the acupuncture treatment (Figure 2).

The acupuncture procedure is as follows: after skin disinfection, needles (diameter, 0.25 mm and length, 15 or 40 mm; Hwatuo, Suzhou, China) will be inserted vertically or horizontally into each point. After eliciting *deqi* (28), the needles will be kept in place for 60 min with manipulation twice for 10–15 s every 20 min.

Patients will undergo 12 sessions of acupuncture treatment for a total of 8 weeks, twice per week in the first 4 weeks and once per week for the last 4 weeks. All acupuncture manipulations will be performed by two trained and licensed acupuncturists.

### Medications

In cases of severe pain for migraineurs, ibuprofen will be allowed as rescue medication. The use of medication will be recorded in detail during the trial.

### Outcome assessment

#### Clinical outcomes

Data acquisition will be performed by independent assessors who have received professional training before the start of the study. Clinical outcomes are as follows:

##### Number of days with migraine and frequency of migraine attacks

Each patient with MwoA will record the number of days with migraine, frequency of migraine attacks, onset time, accompanying symptoms, duration, and methods used to relieve the headache in a headache diary (29, 30) during the trial.

##### Response rate

Response rate is defined as the proportion of patients with ≥50% reduction in the number of migraine days (31), which will be calculated at the end of treatment (week 8) and end of follow-up (week 20).

##### Average visual analog scale (VAS)

Average visual analog scale scores for pain will be used to assess changes in headache intensity from the baseline to the end of follow-up (32). Scores will be recorded every 4 weeks to evaluate the severity of headache for the previous 4 weeks.

##### Evaluation of emotional state and usage of ibuprofen

The emotional state of patients will be assessed using the *Zung* Self-rating Anxiety Scale (SAS) and Self-rating Depression Scale (SDS) at weeks 0, 4, 8, 12, 16, and 20. The use of ibuprofen during the trial will be recorded every 4 weeks.

#### Multimodal MRI data acquisition

MRI data will be acquired at week 0 with a 3.0T magnetic resonance scanner (GE Discovery 750; Milwaukee, WI, USA) with a 16-channel phase-array head coil at the MRI Center of the University of Electronic Science and Technology in China. Participants will be asked to lie in the supine position on the examination bed, stay awake, keep their head still, with eyes closed and ears plugged during the scan.

Three-dimensional T1-weighted (3D-T1) imaging will be performed to obtain high-resolution structural images for each participant with a voxel size of 1 mm^3^, using a spoiled gradient-recalled sequence (repetition time [TR], 6.008 ms; echo time [TE], 1.7 ms; field of view [FOV], 256 × 256 mm^2^; data matrix: 256 × 256). Resting-state blood oxygenation level-dependent (BOLD) signals will be obtained by gradient-recalled echo planar imaging (31 contiguous slices; slice thickness, 5 mm; TR, 2000 ms; TE, 30 ms; flip angle, 90°; FOV, 240 × 240 mm^2^; data matrix, 64 × 64; total volumes, 205).

To detect any white matter abnormalities, diffusion tensor imaging (DTI) will be performed with the following parameters: TR, 8500 ms; TE, 84 ms; data matrix, 128 × 128; FOV, 256 × 256 mm^2^; slice thickness, 2 mm; and 78 continuous axial slices with no gap. Two diffusion-weighted sequences will be acquired using gradient values b = 0 s/mm^2^ and 1,000 s/mm^2^ with diffusion-sensitizing gradients applied along with 64 non-linear directions.

### Data analysis

Collected data will be managed with printed case report forms (CRFs) and only outcome assessors will have access to these data. The research team will be responsible for data organization. Two data entry checks will be performed. The Ethics Committee of the First Affiliated Hospital of Chengdu University of Traditional Chinese Medicine will be supervising this study with a check-in every 3 months and will make the final decision on whether or when to terminate the trial.

#### Clinical data analysis

Clinical outcomes will be analyzed using SPSS v22.0 statistical software (IBM, Armonk, NY, USA). The level of statistical significance (two sided) will be set as 0.05 in all analyses. The per-protocol set was the primary population for efficacy analyses.

All continuous variables will be expressed as means with standard deviations. Categorical variables will be described as numbers and percentages. The Kolmogorov–Smirnov test will first be used to test the distribution of continuous variables. For normally distributed data, the paired-sample *t*-test will be used to evaluate differences within groups; when the data follow a skewed distribution, a non-parametric test will be used.

#### MRI data preprocessing

BOLD functional MRI data will be preprocessed (i.e., slice timing, motion correction, spatial normalization, detrending, and bandwidth filtering) using MATLAB R2018a (Mathworks, Natick, MA, USA) and SPM12 (SPM12; Wellcome Department of Imaging Neuroscience, London, UK; http://www.fil.ion.ucl.ac.uk/spm/) software. After preprocessing, the amplitude of low-frequency fluctuation (ALFF), fractional ALFF (fALFF), regional homogeneity (ReHo), functional connectivity (FC), and other possible brain functional parameters will be analyzed. The 3D-T1 data will be analyzed by voxel-based morphometry using SPM12 and the CAT12 toolbox in MATLAB (33). DTI data will be preprocessed and analyzed using Functional Software Library v4.1.9 (http://fsl.fmrib.ox.ac.uk/fsl/fslwiki/TBSS/). Fractional anisotropy (FA) index and mean diffusivity (MD) maps will be obtained after head action correction, eddy current correction of DTI images, and tensor model fitting.

#### Classification and prediction using machine learning methods

Multivariate pattern analysis (34) will be carried out to classify migraineurs and HCs; this will include feature selection, model construction, and performance evaluation. Principal component analysis (35) will be performed and the searchlight algorithm (36) will be used to select features from multimodal parameters of brain regions, such as gray matter volume (GMV), ReHo, ALFF, fALFF, FA, and MD. The linear kernel support vector classification will be used to construct the classification model (37), which will be assessed by leave-one-out cross-validation (LOOCV). Clusters with a minimum accuracy of 70% will be identified as meaningful classifying features.

Patients with MwoA will be divided into the response and non-response groups (defined as ≥50% and <50% reduction, respectively, in the number of migraine days end of treatment compared with baseline). Support vector regression will be performed to discriminate between the response and non-response groups using the abovementioned classifying features of brain regions (ie, GMV, ReHo, ALFF, fALFF, FC, FA, and MD) (38). In addition to neuroimaging features, we may combine multidimensional features such as clinical data to build the model. After constructing the prediction model for patients with MwoA, LOOCV will be performed to evaluate the robustness of the model. A receiver operating characteristic (ROC) curve will be generated, and the area under the curve (AUC, 0–1) will be calculated to assess the power of the model. The performance of the SVR model will be quantified by calculating sensitivity and specificity based on the number of true positives and true negatives.

### Patient safety

Routine examination of blood, urine, and stools; blood biochemical testing (including liver and kidney function); and electrocardiography will be performed for each participant before trial enrollment. Adverse events caused by acupuncture, such as bleeding, hematoma, pain, and needle-related fainting, will be recorded in detail in the CRF. Severe adverse events such as death, life-threatening disability, or the need for hospitalization will be reported to the project leader, the research institute, and the ethics committee within 24 h and will be documented in the CRF.

### Quality control

Diagnostic, inclusion and exclusion criteria will be strictly applied to screen participants. Patients with MwoA and HCs will be matched in age and education, and patients with average headache VAS scores at baseline >3 will be included. Before the study, the acupuncturists will undergo standardized training and will be required to strictly control the manipulation of the acupuncture needle to minimize interindividual differences. MRI data collection will be carried out according to the operating procedures and experimental design. Communication with patients will be controlled to minimize the influence of the environment and other factors.

## Discussion

Previous studies have demonstrated the feasibility and validity of predicting acupuncture treatment outcomes in patients with migraine using machine learning technology based on Fisher's z transformation ALFF (27) and fALFF maps (26). However, multimodal brain imaging features have higher prediction accuracy than any single modal feature (39). Machine learning technologies are useful for extracting key features from large complex data obtained by multimodal neuroimaging. Machine learning approaches such as support vector machines, artificial neural networks, decision trees, and Bayesian networks have been widely used to develop predictive models with effective and accurate decision-making capabilities (26, 27, 34).

Using machine learning methods to screen positive responders to acupuncture treatment for migraine is a novel approach for improving clinical outcomes and identifying specific patients who would most benefit. This study can help to better understand the brain structural and functional features in patients with MwoA that are critical for the analgesic effect of acupuncture treatment.

### Limitation

Acupuncture is an effective evidence-based treatment for migraine. The main strength of this study is identifying patients with migraine who will respond positively to acupuncture treatment based on objective multimodal MRI data. However, there is no sham acupuncture group in this trial because constructing prediction models of sham treatment is of limited value for improving the clinical efficacy of acupuncture.

## Trial status

This trial was registered with the Chinese Clinical Trial Registry (http://www.chictr.org.cn) on January 31, 2021 (registration no. ChiCTR2100042915; protocol version no. 2.0). Patient recruitment began on March 1, 2021, and 15 patients with MwoA have been recruited to date.

## Ethics statement

The studies involving human participants were reviewed and approved by Institutional Review Boards and Ethics Committees of the Hospital of Chengdu University of Traditional Chinese Medicine. The participants provided their written informed consent to participate in this study.

## Author contributions

ZL and FZ conceived the study. ZL, FL, SC, and XZ initiated the study design. SC, XZ, and NJ helped with its implementation. SC, XZ, and HZ drafted the manuscript. JZ, XL, YF, and XH revised the manuscript. All authors contributed to the refinement of the study protocol and approved the final manuscript.

## Funding

This trial was financially supported by the National Natural Science Foundation of China (nos. 81973958, 82225050, and 82205288), Innovation Team and Talents Cultivation Program of National Administration of Traditional Chinese Medicine (no. ZYYCXTD-D-202003), and China Postdoctoral Science Foundation (nos. 2017M610593, 2018T110954, and 20190037). The sponsor plays no part in study design, collection, or management and will not participate in analysis, interpretation of data, writing of reports, and the decision to submit reports for publication.

## Conflict of interest

The authors declare that the research was conducted in the absence of any commercial or financial relationships that could be construed as a potential conflict of interest.

## Publisher's note

All claims expressed in this article are solely those of the authors and do not necessarily represent those of their affiliated organizations, or those of the publisher, the editors and the reviewers. Any product that may be evaluated in this article, or claim that may be made by its manufacturer, is not guaranteed or endorsed by the publisher.
